# Laparoscopic removal of an intrauterine device from the sigmoid colon

**DOI:** 10.12669/pjms.311.6096

**Published:** 2015

**Authors:** Fatih Şanlıkan, Oğuz Arslan, Muhittin Eftal Avcı, Ahmet Göçmen

**Affiliations:** 1 Fatih Şanlıkan, Ümraniye Education and Research Hospital, Istanbul, Turkey.; 2 Oğuz Arslan, Ümraniye Education and Research Hospital, Istanbul, Turkey.; 3 Muhittin Eftal Avcı, Departments of Aegean Obstetrics and Gynecology, Training and Research Hospital, Yenişehir, Izmir, Turkey.; 4 Ahmet Göçmen, Ümraniye Education and Research Hospital, Istanbul, Turkey.

**Keywords:** Dislocated intrauterine device, Laparoscopic surgery

## Abstract

Uterine wall perforation which is commonly seen through the posterior wall of the uterus is the most serious complication of an intrauterine device (IUD). We present a case of laparoscopic removal of an IUD from the sigmoid colon in a 31-years-old female who was admitted to hospital with a history of pelvic pain and abnormal vaginal bleeding for one month. The dislocated IUD was removed from the sigmoid colon of laparoscopic intervention without any complications.

In conclusion, the treatment modality for the removal of a dislocated IUD is possible by laparoscopic surgery in selected patients where the dislocated IUD is accessible.

## INTRODUCTION

The usage of an intrauterine device as a contraceptive method is very common in the world, especially in developing countries. In Turkey, where the fertility rates are slightly higher than the world’s average, the intrauterine device (IUD) is the most commonly used contraceptive method. Due to the high rate of usage of IUD, the complications related to IUD should be considered as an important issue for the gynecologists and obstetricians. IUD’s can cause significant morbidity following migration into the pelvic organs. Uterine wall perforation, which is commonly seen through the posterior wall of the uterus, is the most serious complication.

We present a case of successful laparoscopic removal of an IUD from the sigmoid colon without any complication in a 31-years-old patient.

## CASE REPORT

**Fig.1:**
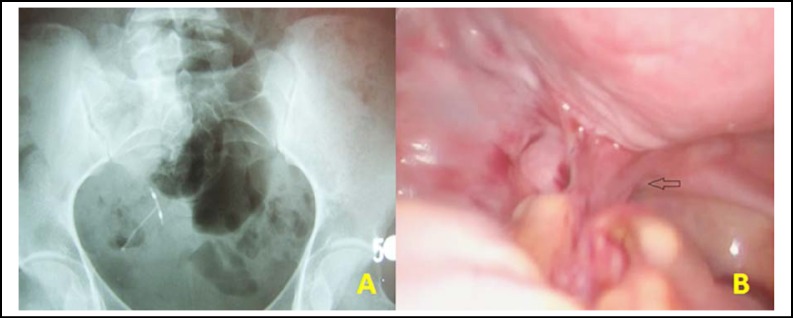
**a- **The X-ray image of dislocated IUD **b**-The laparoscopic view of dense adhesion between the left sacrouterine ligament and the sigmoid colon

**Fig.2:**
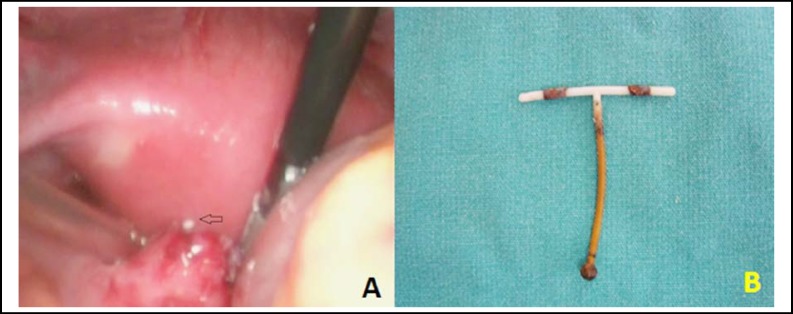
**a**-The image of the tip of the IUD appeared on the serosal surface of the sigmoid colon **b**-The view of removed IUD

A 31-years-old female, gravida 2, para 2 was admitted to our department with a history of pelvic pain and abnormal vaginal bleeding for one month. The patient’s medical history was unremarkable. She had a chronic constipation problem for 4 years but the symptom had become more serious at the time of admission. After her last vaginal delivery, the woman had an intrauterine device inserted six years ago. During the vaginal examination, there was no significant vaginal bleeding and the strings of the IUD could not be identified. The transvaginal ultrasonography revealed that there was no intrauterine device in the uterine cavity. An abdominal X-ray image revealed that the T-shaped IUD with copper was located on the left side of the pelvic wall (Fig.1a). There was no detected organic reason for abnormal vaginal bleeding. The human chorionic gonadotropin test was negative and only the prolactin level high. The patient was informed about the laparoscopic surgery and written informed consent was taken. During the laparoscopic exploration, a suspicious dense adhesion between the left sacrouterine ligament and the sigmoid colon was observed (Fig.1b). The adhesion was then excised and the tip of the IUD appeared on the serosal surface of the sigmoid colon (Fig.2a). The dislocated IUD was removed from the sigmoid colon (Fig.2b). The colonic defect was repaired with intracorporeal single layer suturation to prevent the occurrence of intestinal fistula, infection or other complications related to bowel surgery. Postoperative outcome was uneventful and she was discharged two days after the surgery. The evaluation of the hyperprolactinemia was done after the recovery period and medical treatment was given to the patient. During six months of follow-up, she had no symptom related to the gastrointestinal system.

## DISCUSSION

The IUD is one of the safest, economical and widely used contraceptive methods. Uterine perforation and migration are unexpected complications and occurring in 1.3/1000 users.^1^ Either a part of the IUD can adhere into the uterine wall or entirely involving contiguous pelvic organs, the bladder, appendix or rectum.^2^ Factors related to perforation include design of the device, patient characteristics such as uterine size and position and timing of insertion relative to delivery or abortion. Uterine perforation occurs mostly during insertion and may cause pelvic pain, bleeding from the rectum or vagina. If unrecognized, fibrosis and adhesion formation can occur. Bowel perforation can lead to abscess formation, intestinal ischemia or volvulus.^3^

In a review of the literature, Arslan et al. reported 47 cases of migrating IUD with intestinal penetration which involved the sigmoid colon, followed by the small intestine and rectum.^4^ In some cases, bowel perforation may require surgical intervention ranging from simple closure of the bowel wall to resection of the colonic segment. Inceboz et al. reported a case about laparoscopic removal of dislocated IUD device. The device, which was partially embedded in the sigmoid colon, was removed via laparoscopy; however, because of bowel perforation, they performed laparotomy to open colostomy.^5^ There have been reports in the literature of laparoscopic removal of partially embedded IUDs in the sigmoid colon without any complication.^2^^,^^6^ Minimal invasive techniques should be the main therapeutic approach for IUD related complications and they are increasingly operated with advances in laparoscopy. Reduced tissue trauma, lower postoperative pain and lower risk of pelvic adhesions are known advantages of laparoscopic removal.

On the other hand, laparoscopic removal has had diverse outcomes, with reports of repeat laparoscopy, conversion to laparotomy, in cases which adhesions and perforation are is detected.^7^ In compliance with the literature, we successfully removed an IUD via laparoscopy. The IUD had completely perforated through the sigmoid colon into the lumen and we repaired the defect with intracorporeal single layer suturation. Colonoscopic retrieval may be useful in cases where the device is embedded within the inner part of the wall. Al-Mukhtar et al. reported that colonoscopic retrieval of an IUD perforating the sigmoid colon must be the first choice of therapy.^8^ However, using this method may lead to difficulties if the device is partly embedded in adjacent structures. Without repairing the colonic defect, intraperitoneal contamination from intestinal contents can cause sepsis and need for urgent laparotomy.^9^

In conclusion, the annual vaginal examination of patients who have intrauterine device should be helpful for the checking the location of the IUD. If the strings of the IUD is not visible at external os, uterine perforation should be suspected. Abdominal or vaginal ultrasonography should be used to determine if the IUD is still present in the uterus. If the IUD is not contained in the endometrial cavity, x-ray and computed tomography of the abdomen and pelvis can be useful for diagnosis. In selected patients, rectosigmoid perforations via IUD can be appropriately managed by laparoscopy without any further surgical treatment our case demonstrated that in selected patients, rectosigmoid perforations via IUD can be appropriately managed by laparoscopy without any further surgical treatment.

## Authors’ Contribution:

**Fatih Şanlıkan:** Concept of case, manuscript writing and editing.

**Oğuz Arslan:** Manuscript writing and editing.

**Muhittin Eftal Avcı:** Manuscript writing.

**Ahmet Göçmen:** The operator and manuscript editing.

All authors approved the final manuscript for publication.
